# Comprehensive HIV/AIDS Knowledge and Sexual Behavior among University Students in Ambo, Central Ethiopia: Implication to Improve Intervention

**DOI:** 10.1155/2015/890202

**Published:** 2015-06-09

**Authors:** Zekariyas Sahile, Mulugeta Mekuria, Abenezer Yared

**Affiliations:** ^1^Department of Public Health Officer, Ambo University, P.O. Box 19, Ambo, Ethiopia; ^2^Department of Sociology and Social Work, Ambo University, P.O. Box 19, Ambo, Ethiopia

## Abstract

*Background*. Ethiopia has one of the lowest HIV prevalence rates in East Africa, but there are still more than one million people estimated to be living with HIV in Ethiopia. This study was aimed at assessing the comprehensive HIV/AIDS knowledge and sexual behavior among university students. *Methodology*. A cross-sectional comparative study was done with quantitative and qualitative data collection with a stratified sampling technique. The quantitative data were edited, coded, entered, and analyzed using SPSS software version 20. *Result*. Both comprehensive knowledge of HIV/AIDS transmission and prevention method were higher in the intervention group (75.8% and 48.5%) than comparative group (68.6% and 42.5%) which had a significant difference (*P* < 0.05). Life time sexual intercourse was higher in the intervention group (40.8%) as compared to the comparative group (34.6%). But sexual condom utilization in the past 12 months was higher in the intervention group (73.2%) as compared to the comparative group (56.9%) which had a significant difference (*P* < 0.05). Similarly, history of sexual transmitted disease report was higher in the comparative group (6.3%) as compared to the intervention (4.6%). Among sexual experience respondents in the last 12 months, 32% of them in the intervention and 35.5% of them in the comparative group have had multiple sexual partners. *Conclusion*. The intervention group had higher comprehensive knowledge of HIV/AIDS and condom utilization. But a higher percentage of students were engaged in risky sexual behavior. Therefore, emphasis should be given on designing different strategy to reduce risky sexual behavior and increase comprehensive HIV/AIDS knowledge.

## 1. Introduction

HIV/AIDS is recognized as one of the major public health issues as well as the development problem in Ethiopia since the mid-1980 [1]. The impact of HIV/AIDS goes beyond public health concerns because it primarily affects adult population in the productive and reproductive age groups as such in its endemic stage, it undermines the social and economic structure of developing countries [2].

The national HIV prevalence data in the general population results from the 2005 Ethiopia Demography Health Survey (EDHS) indicates that 1.4% of Ethiopian adults aged 15–49 were infected with HIV and data from 2011 EDHS shows a prevalence of 1.5% ranging from 4.2% in urban population to 0.6% in rural population. Only one-quarter of young women and one-third of young men have a comprehensive knowledge of AIDS, meaning that they know the two major methods for preventing HIV transmission, know that a healthy-looking person can be HIV-positive, and reject the two most common misconceptions about HIV/AIDS [3, 4].

Higher education institutions (HEI) in Ethiopia host young people aged between 19 and 24 years. This age group is often sexually active and among the most vulnerable and at risk of HIV infection. Millions of young people in Ethiopia in general and adolescents and youth in Higher Education Institutions in particular are at high risk of infection from HIV and other sexual and reproductive (SRH) problems. Thus, HIV/AIDS and SRH intervention in HEI are addressed through Comprehensive HIV/AIDS and SRH intervention method that has behavioral, structural, and biomedical interventions [5, 6].

A study in Ethiopia has shown that on average 30% of university students, both male and female, were sexually active and university students are suffering from complications of unsafe sex such us STI and abortion [7]. Qualitative study carried out in HEIs has revealed that inadequate knowledge about HIV and AIDS, substance abuse and addictions, early initiation of sex, and exposure to pornography were reported as a problem [5].

Studies conducted in high school students revealed that students had adequate knowledge about HIV/AIDS despite the risky practices [8, 9]. However, few studies assess comprehensive HIV/AIDS knowledge and relation with risky sexual behavior. One study conducted in eastern Ethiopia showed that only 24.5% in-school adolescents have comprehensive HIV/AIDS knowledge [10]. Even in studies on knowledge of HIV/AIDS and sexual behavior, there was limited information on comprehensive knowledge as an indicator of current HIV/AIDS intervention by Higher Education Institution (HEI). This study was conducted to assess comprehensive HIV/AIDS knowledge and sexual behavior among university students in Ambo, central Ethiopia.

## 2. Methods 

### 2.1. Study Setting

A comparative cross-sectional study was employed using quantitative and qualitative method of data collection in Ambo University main campus, central Ethiopia from January to February 2014.

### 2.2. Sampling

The target population of this study was all regular undergraduate students found in all colleges in Ambo University main campus. The sample size was calculated using two-sample estimation of the difference between two population proportions in order to compare comprehensive HIV/AIDS knowledge and sexual behavior of students between students who had engaged in HEI intervention (second year and above students) and students who had not engaged in HEI intervention (first year students). Since, there was no baseline information the proportion assumed that *P*1 = *P*2 = 0.5. Other assumptions made during the sample size calculation were 5% marginal error (*d*), confidence interval of 95% (*z*(*α*/2) = 1.96), considering sample size correction formula, and 10% nonresponse rate. Based on the above assumption the final sample size for the intervention group was 722 and for the comparison group was 660. For in-depth interview the numbers of interviewees were sought out until conceptual saturation was reached (i.e., until no new concepts were identified in successive interviews).

### 2.3. Sampling Procedure

A stratified sampling technique was used with the strata of students of year study, 1st year students as comparative group and 2nd and above year students as intervention. Number of respondents allocated from each stratum was determined based on the proportion of students who were in each department obtained from university registrar. Then, study participants from stratum were selected by simple random sampling by using lottery technique. For qualitative study purposive sampling technique was employed to identify list of potential in-depth interview participants from students of the campus. During selection of participant consideration was given to class representatives, participants of various HIV/AIDS training, and members of different clubs such as Anti-AIDS Club, Art Club, and youth association for inclusion in study.

### 2.4. Data Collection

Structured, semistructured, and unstructured questionnaires were developed using different literatures [9–12] and pretested before starting data collection in the university students of another campus. The data were collected through self-administered questionnaire and in-depth interview. An interview guide was employed as a data collection tool for in-depth interview.

### 2.5. Measurements

In this research the following operationals were defined as follows.

*Comprehensive knowledge of HIV/AIDS transmission* means knowing HIV transmission method and rejecting two major misconceptions, HIV transmission by mosquito bite and from eating raw meat prepared by a person infected with HIV.

*Comprehensive knowledge of HIV prevention method* means knowing both abstaining from sexual intercourse, condom use, and limiting sex partner to uninfected partners prevents HIV.

*Sexual behavior* means ever have sex, sexual intercourse in the last 12 months, condom utilization in the last 12 months, and multiple sexual partners.

*Intervention group* means students who were in 2nd year and above were considered intervention group.

*Comparative group* means students who were in 1st year were considered as comparative group.

### 2.6. Statistical Analysis

For quantitative method, the data were edited, coded, and entered using SPSS version 20 and descriptive and analytical analysis was conducted. Bivariate regression analysis was employed to identify the significant difference between the intervention and the comparison group. For significance association *P* value less than 0.05 was used as cut off point.

### 2.7. Ethical Consideration

Before the start of the data collection process, ethical clearance was secured from Ambo University, college of medicine and health science ethical review board. The following safeguards were employed to protect interviewees' rights: the research objectives were articulated verbally, oral permission to proceed with the study was received from the interviewees, and the informants were informed of all data collection devices and activities. Moreover, interviewees were assured that stories and quotes will be shared, but no names will be attached to them. Accordingly, no interviewees have been identified in the research report.

## 3. Result

### 3.1. Sociodemographic

A total of 1311 respondents were included in the study, 697 respondents in the intervention and 614 in the comparative group with response rate of 95% and 92%, respectively. Over one-third (37.78%) of respondents in the intervention group and more than one-fourth (26.7%) in the comparative group were females. The mean age of the respondents in the intervention and comparative group was 22 ± SD 1.52 and 20 ± SD 1.29, respectively. The majority of the respondents (79%) in the intervention group and (59%) respondents in the comparative group were Oromo in ethnic group. The median income of respondents was 300 Birr for both groups (Table 1).

### 3.2. Comprehensive HIV/AIDS Knowledge

All of the respondents in both the intervention group and comparative group have heard of HIV/AIDS and six hundred forty-seven (93%) of the senior group have heard of disease that can be transmitted through sexual intercourse (STD). This is higher percentage when compared to junior group; 559 (91.8%) of them have heard disease that can be transmitted through sexual intercourse (STD).

**Figure 1:**
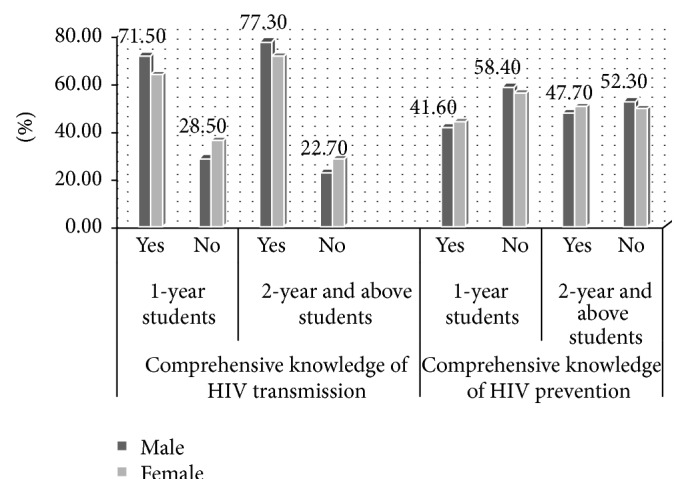
Comprehensive knowledge of the respondents on HIV/AIDS transmission and prevention methods in Ambo University main campus students, January-February 2014.

Of the intervention group respondents, 528 (75.8%) had comprehensive knowledge of HIV transmission mode (77.3% male and 71.3% female). Compared with the situation of the comparative group the percent of both male and female students who had comprehensive knowledge of HIV transmission mode is higher in the intervention group. Of the comparative respondents, 421 (68.6%) of them had comprehensive knowledge of HIV transmission mode (71.5% male and 63.8% female) (Figure 1). On the analysis of bivariate comprehensive knowledge of HIV transmission had a significant association with study groups. The odds of comprehensive knowledge of HIV transmission of the respondents were 1.43 (crude OR 95% CI 1.12, 1.82) times higher in the intervention group as compared to the comparative group.

Similarly, according to the qualitative data obtained from the in depth interview, first year interviewees were able to mention only sexual intercourse as a way of transmission for HIV/AIDS and STDs, while the second year and above student interviewees additionally referred to direct contact of blood, mother to child transmission, sharing of sharp instruments, and the fact that HIV/AIDS is not transmitted through mosquito bites.

Among year one interviewees, one female said, “I do not have detailed knowledge but based on what I hear from Medias, I know that they (STDs) are transmitted sexually and can be cured if noticed immediately,” while another respondent of the same sex said* “there are those (STDs) which come as a result of not using condom. Some people, by thinking that their partner is faithful which no one can be sure of it, may not use condom thereby exposing themselves for those things (STDs).”*

On the other hand, a fourth year female school of law student who participated in various trainings including peer learning, Sister Informing Sister about HIV/AIDS (SISTA), and life skill explained that STDs including HIV/AIDS are* “transmitted when there is blood contact, unsafe sexual intercourse, and from mother to child.”* Besides, a second year male student said that* “there are the so called transmission routes; anything that can have contact with blood can transmit HIV/AIDS.” He also added “They (training providers) have not talked to me about this (whether or not mosquito bites transmit STDs including HIV/AIDS) so far and I am surprised that I have not asked them either. But, I do not think that they are transmitted through mosquito bites because they (STDs) are human diseases.”*

Three hundred thirty-eight (48.5%) students in the intervention group had comprehensive knowledge of HIV prevention methods (47.7% male and 50.5% female). In the situation of the comparative group, the number of both male and female respondents who had comprehensive knowledge of HIV prevention methods was higher in the intervention group. Of the total respondents in the comparative group, 261 (42.5%) of them had comprehensive knowledge of HIV prevention methods (41.6% male and 44% female) (Figure 1). On the analysis of bivariate comprehensive knowledge of HIV prevention method had a significant association with study groups. Being respondents in the intervention group, the odds of comprehensive knowledge of HIV prevention methods were 1.27 (crude OR 1.024, 1.58) times higher as compared to the comparative.

### 3.3. Sexual Behavior

Two hundred seventy-nine (40.8%) of the respondents in the intervention group and 206 (34.6%) of the respondents in the comparative group have ever sexual intercourse. The median age of first sexual intercourse was 18 years in both intervention and comparative groups. Among respondents who have ever sexual intercourse, 75.45% of them in the intervention group and 82.3% of them in the comparative group had sexual intercourse before joining university. Of the total respondents who ever had sexual intercourse, 148 (53%) in the intervention group and 111 (53.8%) in the comparative group had sexual intercourse in the last 12 months. Among respondents who had sexual intercourse in the last 12 months 32% of them in the intervention group and 35.5% of them in the comparative group had multiple sexual partners.

In bivariate analysis, there was association between sexual intercourse in life time and study group (*P* < 0.05). This indicates being student in the intervention group the odds of first sexual practice were 1.30 (95% CI 1.04, 1.64) times higher as compared to the comparative group. However, the association was weak because the confidence interval for OR was nearly 1 (Table 2).

Among the respondents who have had sexual intercourse in the last 12 months, 109 (73.64%) in the intervention group and 66 (59.45%) in the comparative group used condom during sexual intercourse. There was a significant association between study group and condom utilization during the past 12 months (*P* = 0.006). Being students in the intervention group, the odds of condom utilization were 2.06 times higher as compared to student in the comparative group (Table 3).

The history of sexually transmitted disease report in the past 12 months by respondents was lower in the intervention group as compared to the comparative group (4.6% versus 6.3%). However, there was no significant association between study groups and STD history in the past year (*P* value > 0.05).

Findings from the in-depth interview indicated that the comparative group view on the existence of unsafe sex among Ambo University main campus students significantly differs from that of the intervention group. That is, interviewees in their first year of study were not aware of the problem of unsafe sex among students of the campus, as they responded either it is not a problem at all,* “we cannot say it is not”, or “there is nothing I know or have seen.”*

Contrary to the response of the comparative students, the intervention interviewees were well aware of the unsafe sex among main campus students. Besides responding to it as a real problem, a third year male interviewee revealed that* “most of sexual intercourses between students in the campus are unsafe.”* From a point of view that was more of empirically and analytically motivated, a fourth year male student, who is an anti-HIV/AIDS club member and took some HIV/AIDS related trainings, articulated,

I may not put it clear, but there will be unsafe sex among students of the campus. If they have sex without condom, they will be exposed to various infections. And, the evident problem of STDs including HIV/AIDS among the campus students implies that they engage in unsafe sexual practices.

Year one in depth interviewees reported that STDs cannot be considered as problem threatening Ambo University (AU) main campus students. Consistent with what she thought about HIV/AIDS (that HIV/AIDS is not a problem among AU students), a first year female interviewee repeated that she did not think STDs are problems of the campus students. There was one comparative student, however, who believed STDs are problems among the students. Referring to students' failure to use available prevention mechanisms as a reason, she explained,

This is even a campus where it is posted “condoms for free.” They even put condoms at the door steps of or around the clinic. Only fifty percent of university students use it and this makes them affected by HIV/AIDS.

On the other hand, all the intervention group interviewees agreed that STDs are serious challenges of AU main campus students. Demonstrating his knowledge on gonorrhea among STDs, its characteristics, and the illness behavior of patients, a third year interviewee even shared the experience of his female friends in relation to STDs, particularly gonorrhea:

From what I saw is…, gonorrhea is one of the problems. There were two female friends of mine in Ambo University. They told me “I have such problem.” I heard from three persons, three female students. And they have such disease (gonorrhea). I predict there may be a lot of girls affected by this disease.

## 4. Discussion 

The study has compared the first year students who had not engaged in higher education HIV/AIDS/SRH intervention with second year and above students who engaged in intervention on comprehensive HIV knowledge and sexual behavior. The overall comprehensive knowledge of HIV transmission and prevention method was higher in the intervention group which had significant association (*P* < 0.005). This indicates that the intervention group had higher comprehensive knowledge of HIV/AIDS transmission and prevention method.

The intervention group students had higher percentage on life time sexual intercourse (40.8% versus 34.6%) and when compared to the comparative group. The result of the intervention group was comparable with the previous study done in the same university in years of 2011 and 2013, where 39% and 42.8% of respondents have ever sexual intercourse, respectively [13, 14]. Students from the intervention group who had sexual intercourse in the last 12 months were comparable in percentage with students from the comparative group (53% versus 53.8%). Among students who had sexual intercourse in the last 12 months nearly one-third from the intervention group and more than one-third from the comparative group had multiple sexual partners.

Higher condom utilization during sexual intercourse in last 12 months was reported in the intervention group as compared to the comparative group (73.2% versus 56.9%). When we compare in a study done at Ambo university in 2011 that higher percentage of condom utilization was observed, 61.3% used condom during last sex [13]. There was significant association between condom utilization during sexual intercourse in the last 12 months and study groups. The intervention groups were significantly higher in utilizing condom as compared to the comparative group (*P* value < 0.05).

Higher history of sexually transmitted disease (STD) in the last 12 months was reported by the comparative group as compared to the intervention group (6.3% versus 4.6%). The one possible reason for the higher prevalence of sexually transmitted disease in the comparative group might be less proportion of the students had utilized condom in the last 12 months as compared to the intervention group. Regular condom promotion and distribution in Ambo University might be contributed to the higher utilization of condom. Half of the respondents (50%) in the intervention group who had STD in the last 12 months had screened and treated in student clinic which is higher percentage when compared to the comparative (30%). Similarly, the intervention group respondents in depth interview were aware that STDs are series problem of AU students, whereas some of the comparative group respondent were aware the problem exists. Since there was no STI screening, it might be leading to possible bias in self-reporting. Additionally, due to absence of baseline information, the difference on comprehensive knowledge and sexual behavior might not be only the result of intervention done in university so far.

## 5. Conclusion 

Based on the result of the assessment the intervention group student had higher comprehensive knowledge of HIV transmission and prevention method as compared to the comparative group respondents. The difference on comprehensive knowledge of HIV transmission and prevention method between study groups had significantly associated. Nevertheless, nearly one-fourth of the respondents in the intervention group and nearly one-third of respondents in the comparative group had no knowledge about HIV transmission and over half of the respondents in the intervention and comparative group had no knowledge about HIV prevention.

Higher percentage of the intervention group respondents have ever sexual intercourse. But, comparable percentage of the intervention group respondents had sexual intercourse in the last 12 months with the comparative group. Nonetheless, higher percentage of condom utilization during sexual intercourse was reported by the intervention group respondents as compared to the comparative (*P* < 0.005). Correspondingly, higher percentage of STD history in the past 12 months was reported by the comparative group respondents as compared to the intervention group.

Hence, the program managers, higher education administers partners, and health care providers working on HIV/AIDS and SRH intervention should emphasis different strategy in order to improve the comprehensive HIV/AIDS knowledge which is one of the indicators of the intervention and reduction of risky sexual behavior.

## Figures and Tables

**Table 1:** Sociodemographic characteristics of the respondents in Ambo University main campus, January-February 2014.

Variables	Comparative group	Intervention group
Sex		
Male	382 (62.2%)	511 (73.3%)
Female	232 (37.78%)	186 (26.7%)
Age		
18–24	608 (99%)	657 (94.3%)
25–30	6 (1%)	40 (5.7%)
Religion		
Orthodox	321 (52.3%)	341 (48.9%)
Catholic	32 (5.2%)	17 (2.4%)
Protestant	153 (24.9%)	150 (21.5%)
Muslim	100 (16.3%)	165 (23.7%)
Other^*∗*^	8 (1.3%)	24 (3.4%)
Ethnic group		
Oromo	362 (59%)	495 (71%)
Amhara	150 (24.4%)	106 (15.2%)
Tigrea	32 (5.2%)	30 (4.3%)
Sidama	22 (3.6%)	21 (3%)
Gurage	6 (1%)	24 (3.4%)
Other^*∗∗*^	42 (6.8%)	21 (3%)
Marital status		
Single	567 (92.3%)	586 (84.1%)
In relationship	41 (6.7%)	85 (12.2%)
Married	6 (1%)	26 (3.7%)
Income		
<300	418 (68%)	514 (73.7%)
>300	196 (32%)	183 (26.3%)

^*∗*^Waqafaata. ^*∗∗*^Siltea, Wolita, kefaa, and so forth.

**Table 2:** Sexual practice of the respondents in Ambo University main campus, January-February 2014.

Study group	Ever had sexual intercourse				Sexual intercourse in the last 12 months		
	Yes	No	*P* value	95% CI OR	Yes	No	*P* value
Comparative group^*∗*^	206 (34.6%)	390 (65.4%)	<0.021 OR 1.30	(1.04, 1.64)	111 (53.8%)	95 (46.1%)	>0.05
Intervention group	279 (40.8%)	404 (59.2%)			148 (53%)	131 (47%)	

^*∗*^Reference category.

**Table 3:** Proportion of condom utilization and sexually transmitted disease in last 12 months in Ambo University main campus students, January-February 2014.

Study group	STD in the past 12 months			Condom utilization in the last 12 months			
	Yes	No	*P* value	Yes	No	*P* value	95% CI OR
Comparative group^*∗*^	39 (6.3%)	575 (93.7%)	>0.05	66 (56.9%)	50 (43.1%)	<0.006 OR 2.06	(1.23, 3.45)
Intervention group	32 (4.6%)	665 (95.4%)		109 (73.2%)	40 (26.8%)		

^*∗*^Reference category.
